# Capacity to Consent to Sex: A Historical Perspective

**DOI:** 10.1093/ojls/gqae029

**Published:** 2024-08-31

**Authors:** Laura Lammasniemi

**Keywords:** legal history, sexual offences law, capacity, sexual consent, age of consent, mental disability

## Abstract

This article provides a historical perspective on capacity to consent to sex. It examines who could make decisions about sex, whose consent mattered and why. The article draws from legal history and from transcripts and testimonies in unreported sexual offence cases in England, heard in the Central Criminal Court in London and the regional assizes between the years of 1918 and 1950. These cases, often involving vulnerable complainants below the age of consent and those with mental disabilities, show that the concept of capacity was neither fixed nor clearly articulated. The article argues that, historically, capacity was not a biological or medical construct, but rather a social one, influenced by notions of class, gender and even eugenic ideals. The article demonstrates that, during this period, sexual offence law enabled social and population control, and that, despite significant legislative advances, capacity remains a fraught concept.

## 1. Introduction

Who is allowed to consent to sex, and whose consent matters? How does a child gain capacity to consent, and can adults lose it? In this article, I will examine these questions in a historical context. Specifically, I will analyse the boundaries imposed upon women’s and girls’ capacity to consent by criminal law, and how those boundaries were interpreted in English criminal courts in the early 20th century, during the decades leading up to the enactment of the Sexual Offences Act 1956.

In this article, I advance an argument that the undefined concept of capacity, as used in criminal courts, was neither a biological nor a medical assessment, but rather a social one, influenced by notions of class, gender and eugenic ideals. To support this argument, I will trace the legal history of capacity in this context and draw extensively from transcripts and testimonies in lower-level sexual offence cases. In so doing, I will build upon existing historical scholarship on sexual offences and show that the law failed vulnerable complainants in two distinct ways.^1^ First, it failed to sufficiently protect vulnerable people against sexual exploitation and harmful sexual activity. Secondly, it failed to protect the autonomy of those with different capacities, often labelled ‘mentally defective’, and prohibited them from engaging in consensual sexual encounters and romantic relationships. This article and the arguments within are drawn from an analysis of nearly 200 case files of sexual offence cases heard in lower-level courts,^2^ namely the Central Criminal Court in London and the assizes outside London, in the years 1918–50.^3^ Of these 200 cases, 135 were accessed through a privileged agreement with the Ministry of Justice. In line with that agreement, all names, locations, dates and identifiable data have been removed, and pseudonyms are used throughout.^4^ These cases represent a small fraction of all cases heard in the period, and the files that remain in the archives are fragmented—particularly those from the early years of the 20th century—and consist of partial details of transcripts, testimonies and depositions given at the police station. While record-keeping improved during the interwar years, there are significant gaps in the records; for most cases heard in the period, there were no surviving records beyond names in the Court Books. Where partial details of the case exist, the case details have been triangulated using other available sources—mainly online newspaper archives, along with census and workhouse records—to form a fuller picture of the case or to find the sentence imposed.^5^

The argument and methods used in this article are significant in three ways. First, I demonstrate that—historically—capacity was a crucial, if undefined, tenet in sexual offences law. There is little legal historical scholarship on capacity to consent to sex. Throughout history, capacity and incapacity have been legislated through medical and mental health law, and therefore most scholarship on capacity is focused on this context.^6^ Yet, there is related historical literature on mental disabilities and institutions^7^ and on the age of consent,^8^ both of which touch upon the concept within a criminal context. Out of this broader, rich scholarship on institutions and regulatory regimes on mental disabilities, I will draw predominantly on scholarship on ‘mental deficiency’,^9^ as laws regulating ‘deficiency’ directly interacted with sexual offences law.^10^ By analysing the legal history of the concept and associated narratives in criminal courts, I will demonstrate that, historically, capacity as it was used in the courts was not objectively assessed. Instead, this assessment, both in law and in practice, was clouded by external factors such as social class, gender and the ideals of the eugenics movement. Secondly, by examining a range of previously unanalysed, unreported lower-level cases, I shed light on women’s lived experience of law during the period. Analysis of these lower-level trials, rather than those in the then appellant courts of the Court for Crown Cases Reserved or the Court of Criminal Appeal, gives an insight into how criminal law functioned in practice. This practice was often far removed from set principles in the period. The analysis illustrates the difficult experiences of women and girls within the criminal justice system, and how they attempted to navigate that system. Both these contributions are of wider importance, as it was during this period of the late 19th century and early 20th century when legal principles were solidified, and the conceptual foundations of modern sexual offences laws were laid.

Finally, gaining a better understanding of that legal history is of contemporary relevance, as issues related to capacity are yet to be resolved, despite significant legislative advances in the 2000s. While an assessment of capacity is now built into the language of the law,^11^ there remains ambiguity over its definition, and the extent to which intoxication, power relations or mental disabilities can impact capacity.^12^

This article assesses the concept of capacity in the following three sections. In section 2, I discuss the fragmented laws on sexual offences in the period, to illustrate the importance of capacity to the contemporary sexual offences framework, and its conceptual inconsistencies within that framework. In section 3, I focus on children and young people, arguing that, despite clear boundaries setting a minimum age of consent, working-class girls in particular had to attest to their incapacity. Finally, I focus on prosecutions under the Mental Deficiency Act (1913, and discuss capacity and incapacity in the context of mental disability and institutionalisation. The case analysis in section 4 also illustrates the extent to which criminal courts enforced and opted into social control and eugenics, and the impact this had on the lives of women caught in those regulatory regimes.

## 2. Capacity in the Context of 20th-Century Sexual Offences Law

Historically, sexual offences—whether common law or statutory—were structured around two important concepts: (i) the overpowering of the will of the complainant; and (ii) what in the present day we would call capacity. These were two distinct concepts, assessed separately. For much of the 20th century, force and evidence of resistance during an assault were construed as the most reliable indicators of the overpowering of the will of the complainant, or the absence of consent.^13^*Consent* was historically construed through physicality, in contrast to the present day.^14^*Incapacity*, on the other hand, was something more fundamental and enduring. Absence of capacity meant (or ought to have meant) that consent was irrelevant to sexual offence proceedings, as the complainant was unable to make valid decisions about sex. Despite its significance, capacity was not a coherently articulated concept in 20th-century criminal law. Instead, capacity was legislated on in specific contexts, such as age, mental disability and incest, demarcating boundaries between who was allowed to consent to sex and whose consent was invalid. Nicola Lacey has evaluated the history of capacity in relation to responsibility, and argued that in that context, subjective capacity was not a long-standing tradition in English common law, but rather one that emerged in the 19th century.^15^ Insanity, as a legal concept and as a defence, also emerges in the 19th century; it is not until 1843 that an insanity defence is clearly articulated in common law, in the case of *M’Naghten*.^16^ It is in this context of insanity and individual criminal responsibility that capacity has been traditionally evaluated by the criminal law thinkers, such as Nicola Lacey, Alan Norrie and more recently Arlie Loughnan.^17^ Such scholarship shows that incapacity and mental unsoundness were recognised in criminal law and used to assess culpability. What, then, of the capacity of women and girls, and their ability to make decisions about sex in sexual offence cases? How did complainants lose and (re)gain capacity?

For most of the 20th century, the law on sexual offences was formed by a complex patchwork of common and statutory law.^18^ The common law offence of rape was the cornerstone of that framework, but there were numerous different offences with which the defendant could be charged, following non-consensual sexual intercourse. Rape was a very serious charge, for which the death penalty had only been abolished in 1841; the charge was usually reserved for the gravest and most violent instances of sexual offending. In the 1700s, Sir Matthew Hale defined rape as ‘carnal knowledge of any woman above the age of ten years against her will, and of a woman child under the age of ten years with or against her will’.^19^ A modernised definition of rape as ‘the carnal knowledge of a woman against her will’, and later without her consent, remained in use until the statutory definition of rape as ‘unlawful sexual intercourse without consent’ was created in the Sexual Offences (Amendment) Act 1976.^20^ Instead of rape, the overwhelming majority of sexual offence cases reviewed for this article were charged as less serious statutory offences, such as indecent assault,^21^ procurement,^22^ sexual offences against children,^23^ sexual offences against those with mental disabilities^24^ or against those institutionalised.^25^ The frequent use of these less serious statutory offences was reflective of the systematic undercharging of the offence of rape.^26^

Under the contemporary legal framework on consent and sexual offences, an assessment of capacity is built into the statutory definition of consent^27^ and related supporting sections.^28^ For most of the 20th century, however, this was not the case. As neither consent nor capacity was defined in common or statute law, there was significant ambiguity over the scope of these important terms in courts. Vulnerability and lack of capacity were more commonly recognised in cases of deception—however, without the deceptive element, there were no coherent rules or agreed interpretation of capacity.^29^ The issues associated with compromised capacity in contemporary criminal law (such as alcohol and drug misuse,^30^ extreme external pressures^31^ or power relations^32^) were not routinely considered in courts, even if elements of these were present in the cases. Courts disregarded the impact of power relations upon the complainants’ capacity to consent or withhold consent in cases where the defendant was a teacher, a coach or even a primary carer.^33^

While incest had become a criminal offence in 1908,^34^ the law only applied for blood relatives. In the cases reviewed for this article, there were multiple cases of stepfathers accused of carnal knowledge of girls or young women in their care, where neither issues of capacity nor the power dynamics were acknowledged. The courts were often ill-equipped to acknowledge and address these complex power dynamics between complainants with compromised capacity (such as teenagers) and their primary carers if the cases fell outside the limited exceptions recognised by law. The exceptions where criminal law recognised absence of capacity fell into two broad categories: children; and in a more limited sense, certain groups of women who had or were perceived to have mental disabilities.

Much legislative activity on capacity had historically focused on age, and the lack of physical maturity had been seen as a key indicator of incapacity. Intercourse with a female child under the age of 12 had been made a capital offence in England in 1285.^35^ This early statutory provision setting a minimum age is unusual; most European countries did not have age-of-consent legislation until more recent centuries, instead using biological sexual maturity as the determining factor.^36^ However, the English historical offences were vague, leading to centuries of confusion in the interpretation of the seriousness of the offence, and whether intercourse with a child was a felony or a misdemeanour.^37^ These ambiguities were clarified in the 19th century, after the law was reformed several times,^38^ and then finally, in 1885, the Criminal Law Amendment Act set the age of consent at 16. The CLAA 1885 maintained the distinction between a felony charge for carnal knowledge of girls under the age of 13^39^ and a misdemeanour for those under the age of 16.^40^

While the most clearly articulated rules on capacity related to age, physical maturity was inconsistently recognised. In the early 20th century, the minimum age for marriage with parental consent defined by common and canon law was set at 12 years for girls and 14 years for boys. This low age was tied to reproductive capacity, reflecting the beginning of puberty and the ability to conceive a child.^41^ The minimum age for marriage came within a statutory footing only in 1929, when the Age of Marriage Act increased the minimum age of marriage to 16 for both girls and boys, with parental consent.^42^ This is notable, as consent to marriage required a low level of intellectual capacity^43^ and included consent to all sexual relations within that marriage.^44^ For decades, then, a girl child was deemed to lack capacity when it came to sex *outside* marriage, but not when it came to marriage or reproduction.

In relation to those with learning difficulties, mental disabilities or severe mental health issues, the rules governing capacity developed during the period of study, but in inconsistent ways that afforded little to no protection to women and girls who lacked an understanding about sexual activity and the ability to make decisions about sex. Instead of protecting individuals with mental disabilities, at the turn of the 20th century, both courts and medical literature framed women with mental disabilities as sexually threatening to men—who might be unable resist them.^45^ This positioning of women and girls with mental disabilities as ‘sexually precocious, possessing “animal instincts”’, as Joanna Bourke argues, allowed the courts to repeatedly diminish sexual harms against those with compromised capacity.^46^ The term ‘animal instincts’ was first articulated in the 1859 case of *Fletcher*, which concerned the rape of a 13-year-old girl with severe learning difficulties. In his direction to the jury, Judge Keating introduced the term ‘animal instincts’, saying that women and girls with mental disabilities ‘might not be able to know right from wrong’, and ‘in some cases they might be consenting parties, through the force of animal instincts’.^47^ In cases where the disability was of such severity that the complainant was unable to understand what was said to her, defendants continued to be convicted without any clear rules of where the boundaries were set.^48^ Despite this flexibility, the introduction of the term ‘animal instincts’ in *Fletcher* meant that incapacity due to mental disability could be used to *signal* consent, rather than indicate the inability to form it.

From a contemporary perspective, the failure of the law to recognise and address issues of capacity impacting those with mental disabilities is notable, as those with learning difficulties are particularly vulnerable to sexual exploitation for a whole range of reasons, from lack of awareness and communication skills to complex dependencies.^49^ Criminal law’s reluctance to even accept that severe mental disability could indicate inability to consent was somewhat mediated by section 5(2) of the CLAA 1885. The section applied in cases of carnal knowledge of ‘any female idiot or imbecile woman or girl’, in circumstances that fell short of rape, if the accused knew the woman or girl was ‘an idiot or imbecile’. This offence was rarely charged, and it was a far less serious offence than rape; it was a misdemeanour, for which the maximum penalty was two years’ imprisonment.^50^

By the early 20th century, issues of capacity for women who had mental disabilities became further complicated, as mental capacity law became infused with eugenic ideals. A powerful and problematic offence was created under section 56 of the Mental Deficiency Act 1913, which criminalised carnal knowledge of a woman who had been certified as a ‘mental defective’. This new offence, discussed in more detail in the final section, aimed to achieve social control of people with mental disabilities; it aimed first and foremost to stop those certified as ‘feeble-minded’ from having children.^51^ To further this aim, sexual intercourse with a woman who had been certified as a ‘mental defective’ became a criminal offence. Section 56 only applied to women who had been certified as such, and made no provision for men and boys who had mental disabilities. Certification was a bureaucratic exercise, as discussed in the final section of this article, that did not necessarily take into account the mental abilities of the woman in question. As it only applied to women who had been certified, the Mental Deficiency Act brought no clarity to sexual offences law on the question of capacity.

## 3. Evaluating Capacity of Children and Young People in Court

Can a child consent to sex, and when does a child become an adult? Despite the seemingly rigid rules on age of consent, discussed above, criminal courts found it extraordinarily difficult to answer these questions.^52^ This section draws extensively from surviving records of the cases heard in the Old Bailey and regional assizes, between the years of 1918 and 1950. The analysis of these cases will show that in the courts, assessments of capacity were clouded by assumptions of gender and social class, with the family circumstances of the young complainants often discussed explicitly as a way to ascertain sexual and physical maturity.

Irrespective of the circumstances of the alleged assault, most sexual offences against children were prosecuted under the CLAA 1885, either under section 4, relating to carnal knowledge of a child under the age of 13, or section 5, dealing with carnal knowledge of a girl above the age of 13 years and under the age of 16 years. The offences under the CLAA 1885, unlike the charge of rape, did not require evidence of force, or that they were committed against the will of the complainant. This is significant, as, when enacted, the CLAA 1885 had not been envisaged as a replacement for the rape charge. Section 5 had been enacted specifically to criminalise men who seduced teenage girls unable to make informed decisions about sex because of their youth.^53^ In practice, such cases did materialise in courts: there were a series of prosecutions throughout the period of study against men of all ages who had formed romantic relationships, often leading to pregnancy, with teenage girls under the age of 16. This provision, when used to prosecute seemingly consensual sexual encounters with young people, was deeply unpopular with the judiciary, some of whom openly slammed the obligation to send ‘young men of good reputation’ to prison.^54^ Despite this, they conceded that, as long as parents voiced their objection to the relationship and reported the offence, their hands were tied. However, such cases did not form the majority of prosecutions under section 5, most of which were cases of forceful and non-consensual carnal knowledge, where the complainant happened to be under the age of consent; and in those cases, a charge of rape could—or should—have been brought. As such, the section 5 offence was used to prosecute a broad range of instances of sexual offending, from sexual relationships between two romantically involved people close in age, to instances of violent rape.

The overuse of the section 5 offence was significant beyond the question of fair labelling. CLAA 1885, section 5 was a misdemeanour for which the maximum penalty was two years’ imprisonment. This was a significantly less serious penalty than that which a defendant would face for rape. Although rarely given, the maximum sentence for rape was life imprisonment. By preserving the rape charge for the most serious instances of sexual offending, often accompanied by evidence of force and resistance, the CLAA 1885 allowed sexual offending against children and teenagers to be dealt with by less serious, statutory offences—implying lesser culpability. The overuse of the section 5 offence was also significant, as the vast majority of complainants in all sexual offence cases in the period were below the age of consent. Therefore, the overwhelming majority of sexual offence cases that could—and, on the facts, should—have been charged as rape were instead charged as less serious, statutory offences under CLAA 1885.

The average age of complainants in all the sexual offence cases reviewed for this article was 15 years. Due to the fragmented nature of criminal records, the average age of complainants across the country cannot be confirmed with any level of certainty. Nevertheless, the low average age of complainants discovered in my study is in line with earlier studies by Louise Jackson and Carolyn Conley, both of whom confirm that adult women were significantly under-represented in sexual offence cases in the Victorian period.^55^ There are several reasons why cases involving adult or older complainants did not reach the courts, not least the various social and legal exclusions that prevented women from bringing claims against their intimate partners.^56^ Secondly, rape myths and presumptions about ‘good victimhood’ were rife,^57^ and the victim’s sexual history—and medical evidence of that history—was openly discussed in court.^58^ The importance placed on virginity decreases in the interwar years, yet even in the 1940s, medical experts routinely testified as to whether the hymen had been broken during the alleged assault. Due to the over-representation of children and young people in sexual offence cases, the questions of capacity and adherence to strict limits of age of consent, therefore, were of the utmost importance.

### A. Social Class and Cross-Examination

Despite seemingly clear legal rules, social class and gender had significant impact on the way capacity and incapacity were viewed. As demonstrated in the sections above, children under the age of 16 did not have capacity to consent to sex in law. Yet this section shows that both the law and the way in which court proceedings were structured allowed that incapacity to come under sustained assessment and attack in courts.

Social class, class dynamics and assumptions about the working classes impacted trial proceedings and discussions on capacity in several ways. The overwhelming majority of complainants of all ages came from working-class families and communities. The girls and female children who were complainants faced class prejudice, in both implicit and explicit ways. There was an implicit assumption that working-class girls below the age of consent had sexual awareness and knowledge, if not rapaciousness, beyond their years. In the 1910s and 1920s, girls from working-class backgrounds were often in service by the age of 14, and younger complainants narrated their daily chores and tasks, paid and unpaid, in their testimonies to the courts. By the 1940s and 1950s, this had changed, and many complainants below the age of 16 were reported to be in school. The fact that working-class girls were in employment, often in domestic servitude of some kind, meant that the working-class childhood was framed differently to middle-class childhood, and working-class girls were seen as more precocious, both physically and sexually.^59^

While technically those under the age of 16 could not have capacity in law, judges and expert witnesses often made assumptions and statements implying the opposite, framing young complainants as willing participants who were actively making decisions about sex based on desire, foolishness or lack of moral fortitude. I have argued elsewhere that in the 19th century, the ‘courts failed to give meaningful protection to working-class girls because the rules of evidence allowed the courts to realize their ingrained gendered biases about the Victorian working-class family, girlhood, and motherhood’.^60^ This remained true well into the 20th century. In case after case, defence counsel attacked girl complainants over their perceived sexual history, their poor upbringing, use of alcohol or unfortunate family circumstances. This is particularly notable in the court environment within which children and young people had to narrate their traumatic experiences—a deeply lonely and unsupportive one. Women were banned from all formal roles within the court until 1919,^61^ and even after that, female relatives or friends who wanted to support the complainant or women who wished to observe trials due to their professional roles could not attend a sexual offence trial unless it was presided over by a sympathetic magistrate or a judge. Therefore, even young children who had been victims of sexual violence were often left without support in court, as the space was cleared of women when ‘sensitive’ topics were being discussed.^62^ In a 1918 case, a nine-year-old victim of sexual assault had been left in the courtroom to give her testimony with no support, ‘trembling with anxiousness’; all women, including her mother and the woman police officer who attended the case, had been told to leave the room before any evidence was heard.^63^ Cross-examination was the principal tool used by the defence to imply adult status and capacity to consent for young complainants. Throughout the period of study, complainants of all ages had to testify to their lack of consent and be cross-examined—despite lacking the capacity to consent in law. Under section 5 of the CLAA 1885, it was a sufficient defence if the defendant had a *reasonable belief* that the girl was above the age of 16. This clause on ‘reasonable belief’ allowed sexual capacity to be assessed in courts, despite superficially clear rules on age of consent.^64^ The very same factors that were used to discredit the complainant’s testimony, such as past sexual experience, use of alcohol and poor family background, were also used to portray her as more precocious and adult-like by the defence counsel.^65^ Therefore, girl complainants continued to be cross-examined on issues of consent and resistance, at times aggressively, to demonstrate to the jury that the girl in fact appeared older and more experienced than her age, and that she indeed did have the *capacity* to consent to sex and initiate it.^66^

In cases where the complainant was under the age of 13 and no reasonable belief clause existed, young children still had to testify in courts. Even complainants below the age of 10 were routinely cross-examined in court—by the defendant himself in the 1900s and 1910s, and later, as counsel become more common, by legal counsel. All victims who were medically well and old enough to talk testified in court, even if they were too young to understand the nature of the oath and therefore could not be sworn in.^67^ Only in one case reviewed for this article did the counsel outright refuse to cross-examine a five-year-old boy, who (in the opinion of counsel) was too young to understand the questions put to him.^68^ The lack of corroborating evidence and concern over the validity of the evidence in this case led to the defendant’s conviction being quashed in a superior court.^69^

At times, cross-examinations allowed class dynamics to be played out unashamedly for all those in the court to see, particularly in cases where the defendant was a well-known figure in the community, such as a doctor, teacher or even police officer.^70^ Despite a well-documented history of violence against women and eyewitnesses to the assault in question, in one 1928 case, the police initially refused to even investigate the allegations of rape against a local doctor. Charges were brought only after the defendant’s own sister intervened on behalf of the complainant.^71^ When the case eventually reached the court, the complainant (a teenage girl) was so aggressively cross-examined over a period of two hours that the police sergeant attending the hearing noted in his letter to his superior that the questioning was too severe, but the ‘girl’s testimony remained unshaken’.^72^ While cross-examinations of particularly young children were rarely this aggressive, some complainants were treated in an appalling manner. In one such case, a 14-year-old girl living with a severe heart condition was cross-examined over several hours about her resistance to the assault, despite an independent doctor testifying that if she had engaged in physical struggle, her heart would fail. The girl was assaulted while cleaning at a boarding house near her home, and fell pregnant as a consequence. She was painted by all witnesses as a shy, physically weak girl, who, due to her heart condition, had to live a sheltered life. When asked about boys with whom she spent her time, the girl responded that she had no friends, and instead spent her time in the evening crocheting. The line of questioning to which she was subjected now appears absurd, but it was no different from that which all the girls of her age had to endure: repeated questions about how friendly she was with the defendant; whether she had received money from him; her previous sexual experiences; whether she had seduced the defendant, etc. The doctor testified that simply walking to a park would have caused the complainant great difficulty, but it did not stop the counsel from suggesting the girl frequently met boys for sex at the nearby park. These cases were not isolated incidents. Some of the cross-examinations were ruthless and extensive, spanning dozens of pages in the archives, attempting to discredit the complainants as promiscuous, frivolous or, in extreme cases, ‘a prostitute’, even in cases where there was no evidence of commercial sex.

This line of questioning, and the painting of working-class girls as sexually rapacious and physically mature, eroded the assumption of incapacity from which those under the age of consent ought to have benefited. The continuous introduction of factors that ought to have been irrelevant—such as parents’ marital status or the complainant’s past alcohol use—demonstrate the degree to which assessment of capacity was intertwined with social and gender norms. When it came to the girl complainants, age did not determine capacity, particularly if the child in question was from a poor background.

### B. Capacity and Gender Difference

The assumptions of early sexual maturity were not simply class-based, but also highly gendered. Unlike the girl child, who was continuously adultified and sexualised, boys were allowed a childlike naivety in their testimonies, and did not have to speak or evidence their lack of consent or capacity.^73^ Whereas even young girls below the age of 10 had to explain the intercourse and their resistance to it in graphic detail, boys of the same age were allowed, if not expected, to use euphemisms, and escaped aggressive cross-examinations. Where use of euphemisms for genitalia, for example, was common for all children below the age of 10, boy complainants were allowed to continue these euphemisms in their teenage years. While girl complainants were expected to address the assault in detail, and have corresponding medical testimonies to support it, this simply was not the case for boys. In these cases, medical reports were brief, and did not have same level of detail on injuries—implying that, for boys, lack of consent did not require a physical struggle, and resistance was not needed to evidence non-consent.^74^ Defence counsel—who were eager to attack girls as promiscuous and press them on financial incentives to agree to sex—rarely had questions for boy complainants beyond the occasional clarification of facts. The discomfort demonstrated by the defence counsel, and their inability to address sexual acts between boys and men, is inescapable. Even in a case where the boy in question had asked for money in exchange for sexual favours, the defence counsel simply ignored it.^75^ This is remarkable, considering the lengths to which the defence would go to portray working-class girls as promiscuous and/or seeking financial favours for sex.

Homosexual acts involving men and boys of all ages were dealt punitively by both law and society at the time. Young boys did not have to testify to their lack of consent at all, as the courts presumed all sexual contact between boys and older men to be non-consensual, and that the boys had no capacity to consent to it. The courts simply accepted that boys of any age lacked the capacity to consent to sex with other men.^76^ This deep discomfort towards acts of homosexuality must be understood in the broader punitive context of the criminalisation of homosexuality in the period. The capital offence of sodomy had been rarely prosecuted in the 19th century, but the death penalty for it was only officially removed from the statute books in 1861.^77^ There had been no executions for this conviction after 1835; however, the early 20th century witnessed an increased criminalisation of homosexuality following the introduction, via the CLAA 1885, section 11, of the new criminal offence of ‘gross indecency’.^78^ Beyond haphazard individual prosecutions, the offence changed the social landscape, as shown by Jeffrey Weeks.^79^ Havelock Ellis, a well-known eugenicist and sexologist, along with other pioneering sexologists of the period, developed congenital and moral weakness theories about homosexuality, and notions of the corruption and degeneration of youth flourished. It is within this highly punitive social landscape, and against the backdrop of increased criminalisation, that sexual offence cases involving boys and young men reached the courts. Homosexuality was deemed socially dangerous and criminal—therefore boys could not have capacity to consent to it.

The examination of cases from the Old Bailey and regional assizes in this section has revealed the complexity surrounding the notion of capacity within the legal framework of sexual offences and the degree to which the concept of capacity was a social construct. Despite clear legal parameters demarcating the age of consent, the courts struggled with determining a child’s ability to consent to sex and with the transition from childhood to adulthood. The cases highlight the intricate intersections of age, gender and social class within the criminal justice system, and the inability of that system to assess capacity in isolation from the broader social context.

The way in which capacity was discussed in courts was impacted by gender as much as social class. Working-class girls were continuously adultified and sexualised, while boys of the same age and class were not. The girl child was propelled into adulthood by her circumstances and unwanted sexual experiences, in a way that boys simply were not. Simultaneously, in itself, the presumption that boys and young men of any age could not consent to sex with those of the same sex was highly problematic, and reflective of the level of social and legal control of homosexuality. From the discussion in this section, it is clear that criminal law failed to clearly and consistently articulate and acknowledge issues with capacity, and systematically address its absence in cases involving children. In the following section, I will move on to consider how the courts understood and applied capacity in lower-level trials, involving complainants living with mental disabilities and impairments.

## 4. ‘Mental Deficiency’: Capacity as Social and Population Control

In 1943, 34-year-old Amy was testifying in a local criminal court, as a reluctant complainant in a sexual offence trial.^80^ In the months leading up to the alleged offence, Amy had been working as a domestic servant for a respectable family in Essex, and was described as a hard worker. She had started a romantic relationship with the defendant, Frank, and soon fell pregnant. Frank had proposed to her when he found out about the pregnancy. Delighted, Amy had accepted the proposal, proudly wearing her engagement ring to the court. Shortly after the pregnancy was discovered by the authorities, Frank was arrested and faced trial for a serious sexual offence: ‘carnal intercourse with a mental defective’.^81^ Following a brief trial, Frank was convicted and imprisoned. Meanwhile, Amy was returned to a mental deficiency colony. Amy had been certified as a ‘mental defective’ under the Mental Deficiency Act 1913 in her teenage years, and she had been detained in mental deficiency colonies and institutions for most of her youth, before being released on licence to work and live in the community. It was in this period she met Frank. The doctor testified that it would have been hard for Frank, also described as a man of lower intellect, to know she was a ‘mental defective’ as the boundaries of ‘feeble-mindedness’ were fine.^82^ Frank’s prosecution was one of many such sexual offence prosecutions in the interwar years, raising complex issues of capacity to consent to sex, social control and the influence of eugenics on criminal law and criminal practice. In this section, I will focus on cases involving adult complainants, and consider when and how adults like Amy might lose the capacity to consent to sex and to family formation.

Despite social norms that privileged marriage, women and girls—particularly from working-class backgrounds—were often presumed to be willing participants in sexual harms committed against them. These presumptions were openly discussed in courts, as demonstrated by the previous sections. This was also true for women who had mental disabilities or serious mental health conditions.^83^ As discussed earlier in this article, at the turn of the 20th century, mental disabilities could still be used to *indicate consent* in courts, rather than prove its absence, since the introduction of the term ‘animal instincts’ in the case of *Fletcher*.^84^ Those with mental disabilities were required to evidence and corroborate their non-consent like all other complainants; medical evidence or assessments of capacity were not routinely included. Narratives of incapacity in sexual offence cases were most commonly introduced to the courts through sympathetic testimonies from family or members of the community, not by doctors. Against a background of dehumanisation of complainants with mental disabilities, a group of unusual cases emerge in the interwar years, going against the grain of prosecutions and trial practices in the era: the prosecution of men for carnal knowledge of a ‘mental defective’ under section 56 of the Mental Deficiency Act 1913. In other words, these were prosecutions of men like Frank, Amy’s fiancé, who found themselves on trial, following what appeared to them to be a consensual, romantic relationship. Against the backdrop of a failure to recognise mental disabilities, and the vulnerability of girls below the age of consent, these cases are highly unusual, and can only be understood in the context within which the offence was enacted: that of the rising influence of the eugenics movement.

### A. Capacity in the Context of Eugenics and Population Control

The concern over or even institutionalisation of those living with mental disabilities was nothing new. For a long time, those labelled ‘lunatics’, ‘idiots’ and ‘imbeciles’ had come under different regulatory regimes, often aimed at confinement.^85^ In the late 19th century, a range of institutions were developed to ‘control, contain, or change nonconformist and problematic behaviour’, as Susan Mumm argues.^86^ The period saw the creation of a range of carceral institutions, such as industrial schools,^87^ district schools,^88^ female penitentiaries,^89^ various reformatories,^90^ asylums^91^ and borstals.^92^ While most of these institutions had ceased to function by the mid-20th century, the far-reaching legacy of the carceral nature of institutions and institutionalisation has been shown in recent influential research by Lucy Series.^93^ The measures adopted in the early 20th century, however, differ from their predecessors due to their sheer scope and reach—and, for the purposes of this article, how they interacted with sexual offences law. To respond to the concern that ‘feeble-minded’ people were not sufficiently cared for or controlled, the Royal Commission on the Care and Control of the Feeble-Minded was established in 1908. The Commission came to the conclusion that there were a growing number of ‘feeble-minded’ people, who posed a threat to themselves and to society. The Commission was concerned that there was insufficient control over these people, that this could lead to ‘crime and misery’ and ‘injury and mischief’, and that these people would transmit their defects to future generations.^94^ Several proponents of eugenics and eugenic societies contributed to the Commission, leading to an open discussion on how ‘feeble-minded’ people needed to be stopped from ‘breeding and weakening the British stock’.^95^ Therefore, eugenics were not merely subtly implied, but also became the very focus of policy and political discussion, even in Parliament.^96^ Gershom Stewart, the MP introducing the precursor to the Mental Deficiency Act, the Feeble-Minded Persons Control Bill, said ‘What we advocate is that these persons [mental defectives] should be segregated in homes and colonies, especially the women during the childbearing age’.^97^ People with cognitive and mental disabilities were construed as a racial and national threat, including by passing on hereditary impairment. There were myths in existence about high levels of fertility and procreation amidst those deemed ‘feeble-minded’, and the Royal Commission concluded that ‘the number of children born to mentally defective parents is abnormally high’.^98^

Recommendations for segregation and institutionalisation were at the core of the Royal Commission’s conclusions, leading to the Mental Deficiency Act 1913, which established ‘mental deficiency colonies’ for the confinement of ‘defectives’. While the mental deficiency homes and colonies started out relatively small, they rapidly grew in size and population, and Sandland estimates that by ‘1939, almost 100,000 individuals were under some form of control imposed by the 1913 Act, many more having passed through the system in the intervening years’.^99^ The Act established a Board of Control for Lunacy and Mental Deficiency, and created a duty on the local authority to ensure that mental defectives were ‘dealt with’, by being placed either in an institution or under statutory guardianship or supervision. The arrangements within the Act were also aimed at managing the sexuality of those deemed feeble-minded and, in particular, to prevent procreation.^100^ Institutionalisation was viewed by many as the politically and socially acceptable solution to the eugenicists’ concerns, and preferable to sterilisation.^101^ Despite more focused campaigns in favour of sterilisation in the 1930s, this never came to fruition because of concerns over sexually transmitted diseases, and whether separating sex from reproduction might leave sterilised individuals vulnerable to sexual exploitation.^102^ To curtail reproduction, all certified women were prevented from having romantic or sexual relationships, and if in an institution, they were physically segregated from men. In other words, all certified women lost the capacity to consent to sex upon certification.

It is against this backdrop of eugenics and the social control of people with mental disabilities that a new sexual offence was also created: carnal knowledge of a woman certified under section 56 the Mental Deficiency Act 1913.^103^ Section 56 of the MDA 1913 made it a criminal offence to have ‘unlawful carnal knowledge of any woman or girl under care or treatment in an institution or certified house or approved home, or whilst placed out on licence therefrom or under guardianship under this Act’. One of the explicit aims of the carnal knowledge section was to protect those with mental disabilities from men who might exploit them sexually. On the surface, this was a good thing, as women who had mental or other disabilities were more vulnerable to exploitation. However, the scope and application of the law went well beyond the protection of women and girls. This section specifically and only applied to women and girls who had been certified as mentally defective, and had no impact on women with mental or other disabilities who had not been certified. Nor did it apply to men who had been certified as a ‘mental defective’, although men could also be vulnerable to exploitation and abuse. The section, therefore, had little to do with protecting individuals with limited or compromised capacity from unwanted sexual advances. The MDA 1913 and its enforcement embodied the eugenics movement’s ideals, and aimed first and foremost to stop women deemed ‘feeble-minded’ from bearing children.^104^

### B. Certification and Social Control

As shown above, the MDA 1913 was brought about by eugenic ideals. It also allowed for social and sexual control of young women who lacked financial and familial support. In an era when sexual offending and sexual harms were not sufficiently recognised by the criminal justice system, section 56 MDA 1913 was an unusually strongly worded offence. Providing the complainant had been certified as a ‘mental defective’, carnal knowledge or attempted carnal knowledge was sufficient basis for liability, unless the defendant could ‘prove that he did not know, and had no reason to suspect, that the woman or girl was a defective’.^105^ A complainant’s consent was irrelevant to the offence. The absolute denial of capacity from women in these cases was remarkable, as a diagnosis of ‘feeble-mindedness’, particularly for women, was not simply a medical fact or assessment, but also a social one. As Thomson has shown, mental deficiency covered a wide range of mental disabilities and conditions, and factors like hearing impairment, dyslexia and even left-handedness could contribute towards a diagnosis; that definition was later expanded to include injuries sustained after birth.^106^

This broad definition of ‘mental deficiency’ is of particular significance when examining cases of ‘carnal knowledge of a mental defective’. In the cases that reached the courts, there were undoubtedly some women who had been victims of (sometimes sustained) sexual abuse, and who were unable to understand and articulate the harm they had experienced. There were also women whose vulnerabilities had been exploited, and they had been groomed for sexual relationships and encounters. Yet, drawing from their testimonies to the courts, some complainants did not fit neatly into these categories. In the trial transcripts, some women articulated in the strongest terms their desire for the sexual or romantic relationship in question, as well as an express desire to leave the institution and live with the defendant. Some expressed no desire to have romantic relationships, but nevertheless unequivocally articulated their consent to sex. Whether any of the women complainants had capacity to consent to sex, as the concept is currently understood, is impossible to ascertain retrospectively from court documents—yet the absence of any real discussion of capacity in courts was notable.

Certification, and by extension denial of capacity, was not simply a physiological assessment. Pamela Cox’s research on the Waifs and Strays Society (an organisation that worked with delinquent or neglected children in the 1920s) shows that social factors at times outweighed physiological factors in certification decisions.^107^ Cox traced the lives of several individual girls who were under the care of the Waifs and Strays Society and found that those girls who were too difficult to manage or were very unlikely to get a position in domestic service were the most likely to be certified. Some girls who had been considered to be sexually deviant were also certified, and, in some cases, insubordinate behaviour could count as evidence of ‘feeble-mindedness’. Cox’s assessment is confirmed by local authority reports at the time. In one county council, an industrial school inspector criticised the fact that the ‘majority of the girls’ at Dovecot (a special industrial school for ‘mental defectives’) were the ‘difficult cases in the schools or homes from which they have been sent’.^108^ In other words, some girls were certified as ‘mental defectives’ simply for being too poor, having no familial support, and being too ‘difficult’ to work in domestic service.

Similarly to Janet Walmsley’s study on the Bedfordshire Mental Deficiency Committee, I found that perceived sexual promiscuity and lack of ‘moral control’ had also been a factor in certification decisions.^109^ In one case, the complainant (who was on the run from a mental deficiency institution at the time of the alleged assault) had been certified 15 years earlier, after falling pregnant out of wedlock in her teens.^110^ At the time of the offence, the complainant had escaped from an institution run by a Catholic charity—known for committing women on moral grounds and those who had illegitimate children—and there was a suggestion that she had engaged in prostitution in her teenage years.^111^ Women, therefore, could be declared to be ‘mental defectives’ and institutionalised for a whole range of reasons. Certification meant that the woman lacked capacity in law, but it did not necessarily mean that she was unable to understand or desire sexual or romantic relationships.^112^

Once women were certified, they could be incarcerated for decades, some all their lives, with no legal right to petition against continued detention.^113^ The Board of Control exercised an enormous degree of control over women and men who had been certified as ‘mental defectives’, denying any resemblance of capacity or autonomy. The decision to certify, influenced by class, poverty and notions of morality, had profound long-term impact. The severity of the certification decision was particularly important, as certification replaced any genuine assessment of individual capacity. The certification process—and, by extension, criminal courts—made no distinction between complainants who had transgressed social and sexual norms and those who exhibited mental disabilities.

### C. Punishing Motherhood

All the cases of carnal knowledge with ‘a mental defective’ that I reviewed were brought after the complainant fell pregnant—the very thing the MDA 1913 was enacted to prevent. Many of the complainants in the sexual offence cases reviewed for this article were under certification but released on licence from the institution where they had been living, as a form of community care. Licences allowed men and women to live in the community, with relatives, and to engage in employment, rather than live in an institution or mental deficiency colony. The licences were regularly reviewed, either quarterly or every six months, and if the women broke the rules of their licence, were unco-operative or left their employment, their licence was revoked and they were returned to the institution.^114^ The licence documents were included in the court papers, and all the licences included a term that the women were not allowed to form an attachment with members of the opposite sex, and that any signs of such attachment must be reported to the local authority and the authorising board and doctor. Women who were released on licence were acutely aware that evidence of any sexual or romantic relationships would lead to their licence being revoked and having to return to the institution. In their testimonies, all complainants emphasised how important not falling pregnant was for them, and how they had been explicit about this importance, at times expressing deep disappointment or anger at the defendant for not having taken precautions to prevent pregnancy. The fears of women were realised, as in each case the complainant had been reinstitutionalised by the time of the trial, and gave the institution as her address.^115^

These prosecutions were unusual in how punitive they were, to both the complainants and the defendants. In some of the cases, the defendant and complainant had clearly formed a serious relationship. If the complainant expressed a desire to continue the relationship or marry the defendant, the institution in question had to give permission. In the case of Amy, discussed at the beginning of this section, both she and the defendant expressed a desire to marry and start a family.^116^ Their request was denied; the doctor of the institution in which Amy was incarcerated stated to the court that ‘the marriage of a person defective detained in an Institution would be very remote, and only with the sanction of the Board of Control’.^117^ Instead, the defendant was imprisoned and Amy was reinstitutionalised.^118^ The prosecutions and aftermath were often far more punitive towards the alleged victim than they were to the accused. If convicted, the defendant spent a maximum of two years in prison, whereas the complainants were often held for decades, and sometimes all their lives, in these carceral institutions. By denying capacity to consent to sex, these women were also denied family life, the chance to care for their own children and the chance to live outside the confines of an institution. These cases, and their profoundly punitive nature, show how deeply intertwined eugenics and social control was with institutionalisation in this context.

Amy’s case is emblematic of many others who were denied the right to make decisions about sex and reproduction under similar circumstances during the interwar period. These prosecutions under the MDA 1913 raise profound questions about capacity, vulnerability and social control, and the role that sexual offences law played in facilitating that control. Despite ostensibly aiming to protect those with mental disabilities from sexual exploitation, the enforcement of the MDA 1913 in courts resulted in the denial of sexual capacity and consent from women like Amy. The MDA 1913, steeped in eugenics and social control, facilitated the incarceration of those deemed a ‘mental defective’, without the right to challenge that incarceration.

## 5. Conclusion

The key finding of this article is that, historically, the ill-defined concept of capacity allowed sexual offences law to be used as a tool for social and population control, and that criminal courts enabled that control. As a consequence, the law failed to protect both vulnerable people against sexual exploitation and the right of people with atypical capacities to a sexual life and romantic experiences. To evidence this finding, I have drawn from legal history, court transcripts and testimonies in nearly 200 lower-level sexual offences in the period leading up to the enactment of the first dedicated Sexual Offences Act in 1956. The 1956 Act made no significant changes to any of the laws discussed, including the MDA 1913,^119^ and resolved none of the issues raised in this article. The issues discussed within the article have, therefore, been persistent and operative for much of the 20th century.

Capacity remains a fundamental tenet of sexual offences law to this day, demarcating the boundaries that determine who is allowed to consent to sex and whose consent is valid. Capacity, as we understand it in the present-day criminal justice system, requires an individual assessment in cases of disability; importantly, incapacity for most adults is not an enduring state of being. In the words of Lady Hale in *R v C*, a case involving a complainant living with schizo-affective disorder, ‘one does not consent to sex in general. One consents to this act of sex with this person at this time and in this place.’^120^ This individualist approach is not extended to children and young people, and blanket age limits, identical to the CLAA 1885, remain in place. This individualised assessment in cases of mental capacity in adults is a notable departure from legal history, as discussed throughout this article. While mental capacity in the context of age and ‘mental deficiency’ had defined rules, an overall definition of incapacity did not exist, nor did consistent approaches to it. Even though it was within the courts’ power to consider incapacity, they failed to address the broader spectrum of factors influencing capacity, such as mental disabilities, power dynamics and societal perceptions.

Despite significant legislative, terminological and social advances, and a much better understanding of mental capacity, not all issues identified in this article are resolved. As reform of sexual offences law began in the early 2000s, the Law Commission concluded that consenting to sex is ultimately different from other decisions in civil law, as ‘it is perceived to be a visceral, rather than a cerebral, process of decision-making’, and so simplifying or adapting civil law tests to criminal law and sexual offences law would be fraught with difficulty, and may even be inappropriate.^121^ Capacity was an important consideration—yet not including a *definition of capacity* in the reform was a deliberate choice.^122^ The Sexual Offences Act 2003, the law that followed a holistic review of sexual offences law, does explicitly address various circumstances where capacity is presumed to be absent or compromised. These include circumstances where the complainant is below the age of consent, asleep or unable to communicate their consent due to disability.^123^ The Act also addresses caring and familial relationships and positions of trust.^124^ Amidst all these exhaustively defined situations, where capacity is perceived to be compromised and consent is presumed absent, the only attempt to define incapacity can be found in sections governing offences committed against persons with a mental disorder that impedes choice.^125^

Despite major changes and clarifications to law and language, fundamental questions remain unanswered around intoxication, imbalances of power and even appropriate limits of age.^126^ To explain contemporary issues, we should look to history to better understand why those questions remain unresolved, and to better understand how deeply intertwined social and gendered perceptions still are with the delicate boundaries set in sexual offences law, and with attempts to enforce those boundaries.

## Footnotes

1 Joanna Bourke, *Rape—A History from 1860 to the Present* (Virago 2007); Joanna Bourke, *Disgrace: Global Reflection on Sexual Violence* (Chicago UP 2022); Victoria Bates, *Sexual Forensics in Victorian and Edwardian England: Age, Crime and Consent in the Courts* (Palgrave Macmillan 2016); Garthine Walker, ‘Rereading Rape and Sexual Violence in Early Modern England’ (1998) 10(1) Gender & History 1; Shani D’Cruze, *Crimes of Outrage: Sex, Violence and Victorian Working Women* (reprint, Routledge 2003); Julia Rudolph, ‘Rape and Resistance: Women and Consent in Seventeenth-Century English Legal and Political Thought’ (2000) 39(2) Journal of British Studies 157; Antony E Simpson, ‘Vulnerability and the Age of Female Consent’ in GS Rousseau and Roy Porter (eds), *Sexual Underworlds of the Enlightenment* (Manchester UP 1987) 182.

2 I have reviewed all available files, cases and appeals in the national archives that include the terms ‘rape’, ‘carnal knowledge’, ‘indecent assault’ and ‘procurement’. Furthermore, voluntary organisations that worked with victims of sexual violence, and selected ecclesiastical records and those of homes for unmarried girls in London have been included in the review (London Metropolitan Archive, Women’s Library).

3 Analysis begins in 1918 to exclude the First World War, as cases during the period are not demonstrative of peacetime due to increased regulation of sexual behaviour in the period, see Philippa Levine, *Prostitution, Race and Politics: Policing Venereal Disease in the British Empire* (Routledge 2003); Angela Woollacot, ‘“Khaki Fever” and Its Control: Gender, Class, Age and Sexual Morality on the British Home Front in the First World War’ (1994) 29 Journal of Contemporary History 325.

4 For both consistency and ethical considerations, I have applied the same level of anonymisation to all cases, irrespective of whether they were accessed through privileged agreement.

5 Reporting restrictions become stricter, particularly in child sexual abuse cases, after 1918 after which reports become more euphemistic. On politics of legal history and newspapers, see Adrian Bingham, ‘“It Would Be Better for the Newspapers to Call a Spade a Spade”: The British Press and Child Sexual Abuse, c 1918–90’ (2019) 88 History Workshop Journal 89; Daniel Grey, ‘“Monstrous and Indefensible”? Newspaper Accounts of Sexual Assaults on Children in Nineteenth-Century England and Wales’ in M van der Heijen, M Pluskota and S Muurling (eds), *Women’s Criminality: Patterns and Variations in Europe, 1600–1914* (CUP 2020) 189–205; Kim Stevenson, ‘Outrageous Violations: Enabling Students to Interpret Nineteenth Century Newspaper Reports of Sexual Assault and Rape’ (2014) 4(1) Law, Crime and History 36.

6 There is a wealth of scholarship on this, see Lucy Series, *Deprivation of Liberty in the Shadows of the Institution* (Bristol UP 2022); Janet Weston, ‘Managing Mental Incapacity in the 20th Century: A History of the Court of Protection of England & Wales’ (2020) 68 Int’l JL & Psychiatry 101524; J Weston, ‘Citizenship, Vulnerability, and Mental Incapacity in England, 1900–1960s’ (2019) 63(3) Medical History 270; Clive Unsworth, ‘Law and Lunacy in Psychiatry’s Golden Age’ (1993) 13 OJLS 479.

7 Susan Mumm, ‘“Not Worse than Other Girls”: The Convent-Based Rehabilitation of Fallen Women in Victorian Britain’ (1996) 29(3) Journal of Social History 527; Paula Bartley, *Prostitution Prevention and Reform in England, 1860–1914* (Routledge 1999).

8 Matthew Waites, *The Age of Consent: Young People, Sexuality and Citizenship* (Palgrave Macmillan 2005); Laura Lammasniemi, ‘“Precocious Girls”: Age of Consent, Class and Family in Late Nineteenth-Century England’ (2020) 38(1) LHR 241; Caroline Derry, ‘Lesbianism and Feminist Legislation in 1921: The Age of Consent and “Gross Indecency between Women”’ (2018) 86 History Workshop Journal 245; Kim Stevenson, ‘Not Just the Ideas of a Few Enthusiasts: Early Twentieth Century Legal Activism and Reformation of the Age of Sexual Consent’ (2017) 14(2) Cultural and Social History 219.

9 The terminology of these regulatory regimes is deeply problematic, so when discussing mental disabilities I follow the terminology set out by Lucy Series. Recognising the importance and failings of terminology, Series (n 6) uses the term ‘mental disabilities’ in relation to people with mental, psychosocial, developmental and other disabilities but historical terms of ‘deficiency’ and ‘lunacy’ when discussed in that historical context. These terms are used in quotation marks throughout, to note that they were neither biological nor medical. For analysis of the language and its complexities, see Patrick McDonagh, CF Goodey and Tim Stainton, *Intellectual Disability: A Conceptual History, 1200–1900* (Manchester UP 2018).

10 Matthew Thomson, *The Problem of Mental Deficiency: Eugenics, Democracy, and Social Policy in Britain c 1870–1959* (Clarendon Press 1998); GR Searle, *Eugenics and Politics in Britain, 1900–1914* (Noordhoff International Publishing 1976); Jane Carey, ‘The Racial Imperatives of Sex: Birth Control and Eugenics in Britain, the United States and Australia in the Interwar Years’ (2012) 2195 Women’s History Review 733; Pauline MH Mazumdar, *Eugenics, Human Genetics, and Human Failings: The Eugenics Society, Its Sources and Its Critics in Britain* (Routledge 1992); Sarah Wise, *The Undesirables: The Law that Locked Away a Generation* (Oneworld Publications 2024).

11 Sexual Offences Act 2003, s 74.

12 Sharon Cowan, ‘The Trouble with Drink: Intoxication, (In)capacity, and the Evaporation of Consent to Sex’ (2008) 41(4) Akron L Rev 899; Alex Ruck Keene and Allegra Enefer, ‘What Place “Capacity” in the Criminal Law Relating to Sex Post JB?’ (2022) 85 Int’l JL & Psychiatry 101843.

13 For discussion on force, see William Blackstone, *Commentaries on the Laws of England*, vol 4; Sir Matthew Hale, *Hale’s Pleas of the Crown* (17th century, University of Manchester Library) 627.

14 Sexual Offences Act 2003, s 74.

15 Nicola Lacey, ‘In Search of the Responsible Subject: History, Philosophy and Social Sciences in Criminal Law Theory’ (2001) 64 MLR 350; Nicola Lacey, ‘Character, Capacity, Outcome: Toward a Framework for Assessing the Shifting Pattern of Criminal Responsibility in Modern English Law’ in Markus D Dubber and Lindsay Farmer (eds), *Modern Histories of Crime and Punishment* (Stanford UP 2007) 14–41.

16
*M’Naghten* (1843) 10 Cl & Fin 200. See also the Criminal Law Commissioners Reports 1833–1845, 7th Report 1843.XIX.1 (Digest of the Criminal Law) 19–20, 127. For analysis of the reports more broadly, see Lindsay Farmer, ‘Reconstructing the English Codification Debate: The Criminal Law Commissioners, 1833–45’ (2000) 18(2) LHR 397.

17 Nicola Lacey, ‘Responsibility and Modernity in Criminal Law’ (2001) 9(3) Journal of Political Philosophy 249; Alan Norrie, *Crime, Reason and History: A Critical Introduction to Criminal* Law (Butterworths 2001); Arlie Loughnan, *Manifest Madness Mental Incapacity in the Criminal Law* (OUP).

18 Lindsey Farmer, *Making the Modern Criminal Law: Criminalization and Civil Order* (OUP 2016).

19 Hale (n 13) 627.

20 Sexual Offences (Amendment) Act 1976, s 1. The full wording is as follows: ‘unlawful sexual intercourse with a woman who at the time of the intercourse does not consent to it; and (b) at that time he knows that she does not consent to the intercourse or he is reckless as to whether she consents to it’. Consent remained undefined until Sexual Offences Act 2003, s 74.

21 Offences against the Person Act 1861, s 52.

22 Criminal Law Amendment Act (CLAA) 1885, ss 2 and 3. For analysis, see Laura Lammasniemi, ‘Trafficking, Rape, or Deceptive Sex? A Historical Examination of Procurement Offences in England’ (2023) 32(4) Social & Legal Studies 499; Chloe Kennedy, *Inducing Intimacy* (CUP 2024).

23 CLAA 1885, ss 4 and 5.

24 CLAA 1885, s 5(2).

25 Mental Deficiency Act 1913, s 56.

26 For undercharging and rape in courts in early modern Britain, see Anna Clarke, *Women’s Silence, Men’s Violence: Sexual Assault in England 1770–1845* (Routledge 1987); Antony Simpson, ‘The “Blackmail Myth” and the Prosecutions for Rape and Its Attempt in Eighteenth-Century London: The Creation of a Legal Tradition’ (1986) 77 J Crim L & Criminology 101; Martin J Wiener, *Men of Blood: Violence, Manliness and Criminal Justice in Victorian England* (CUP 2004).

27 Sexual Offences Act 2003, s 74.

28 The Sexual Offences Act 2003, ss 75–6 lists a set of circumstances where the complainant is presumed not to have consented, such as involuntary intoxication and being unconscious.

29
*R v Flattery* (1877) 2 QBD 410; for a broader analysis of deception, see Kennedy (n 22). CLAA 1885, ss 4 and 5.

30
*R v Bree* [2007] EWCA Crim 804.

31 Extreme poverty and deprivation, *R v Kirk* [2008] EWCA Crim 434; continuum of exploitation and grooming *R v Ali* [2015] EWCA Crim 1279.

32 Sexual Offences Act 2003, ss 16–24.

33 Crim 1/XXX (X489); CRIM 1/2111. All case names and archive references are anonymised hereafter in line with privileged agreement with the National Archives.

34 Punishment of Incest Act 1908. For analysis, see Kim Stevenson, ‘“These Are Cases Which It Is Inadvisable to Drag into the Light of Day”: Disinterring the Crime of Incest in Early Twentieth-Century England’ (2016) 20(2) Crime, Histoire & Sociétés/Crime, History & Societies 31.

35 Statutes of Westminster I and II.

36 Helmut Graupner, ‘Sexual Consent: The Criminal Law in Europe and Overseas’ (2000) 29(5) Archives of Sexual Behaviour 415.

37 Simpson, ‘Vulnerability’ (n 1) 180–205; Rudolph (n 1).

38 The Offences against the Person Acts of 1828, 1861 and 1875 each make amendments.

39 CLAA 1885, s 4.

40 CLAA 1885, s 5.

41 1868 Chelmsford Royal Commission on the Laws of Marriage (HM Stationery Office 1868); for discussion, see Stephen Cretney, *Family Law in the Twentieth Century* (OUP 2005) 40.

42 Laura Lammasniemi and Kanika Sharma, ‘Age of Marriage Act 1929’ in Rosemary Auchmuty, Erika Rackley and Mari Takayanagi (eds), *Women’s Legal Landmarks in the Interwar Years: Not for Want of Trying* (Hart Publishing 2024).

43 Ezra Hasson, ‘Capacity to Marry: Law, Medicine and Conceptions of Insanity’ (2010) 23(1) Social History of Medicine 1.

44 Rape within a marriage was only recognised in 1991, *R v R* [1991] UKHL 12.

45 Ralph Sandland, ‘Sex and Capacity: The Management of Monsters?’ (2013) 76(5) MLR 981. For contemporary discussion on sexuality and mental defectives, see Henry Maudsley, *Body and Mind: An Inquiry into Their Connection and Mutual Influence, Specially in Reference to Mental Disorders, Being the Gulstonian Lectures for 1870, Delivered Before the Royal College of Physicians* (Macmillan 1870) 46–7. On punishing women, including those with mental disabilities, for sexual transgression during the First World War, see Laura Lammasniemi, ‘Regulation 40D: Punishing Promiscuity on the Home Front during the First World War’ (2017) 26(4) Women’s History Review 584.

46 Joanna Bourke, ‘“Animal Instincts”: The Sexual Abuse of Women with Learning Difficulties, 1830s–1910s’ (2020) 29(7) Women’s History Review 1201.

47
*R v Fletcher* (1859) 8 Cox Crim Cas 131, 134 (Ct Crim App). This approach was affirmed in *R v Fletcher* (1866) 10 Cox Crim Cas 248.

48
*R v Barratt* (1871–1874) 12 Cox Crim Cas 498.

49 Bourke, ‘Animal Instincts’ (n 46); Marita P McCabe, Robert A Cummins and Shelley B Reid, ‘An Empirical Study of the Sexual Abuse of People with Intellectual Disabilities’ (1994) 12(4) Sexuality and Disability 297; Michelle McCarthy, ‘Sexual Experiences of Women with Learning Difficulties in Long-Stay Hospitals’ (1993) 11(4) Sexuality and Disability 278; B McCormack, ‘Sexual Abuse and Learning Difficulties’ (1991) 303(6795) British Medical Journal 143.

50 While the offence existed, I did not come across any prosecutions under the section in the case files of the 19th or early 20th centuries.

51 Harvey G Simmons, ‘Explaining Social Policy: The English Mental Deficiency Act of 1913’ (1978) 11(3) Journal of Social History 387.

52 For a broader analysis of age of consent, see Waites (n 8).

53 Lammasniemi, ‘Precocious Girls’ (n 8) 241.

54 National Vigilance Association Case Files, 4NVA/XXX (X12) (held at Women’s Library, LSE), anonymised in line with privileged agreement with Women’s Library.

55 Louise Jackson, *Child Sexual Abuse in Victorian England* (Routledge 1999); Carolyn Conley, *The Unwritten Law—Criminal Justice in Victorian Kent* (OUP 1991).

56
*R v Clarence* [1888] 22 QBD 23.

57 In contemporary scholarship, there has been a significant volume of academic scholarship that discusses these myths. See eg David J Hayes, ‘Are Rape Myths “Myths”?’ (2024) 44(1) OJLS 156; E Daly and others, ‘Myths About Myths? A Commentary on Thomas (2020) and the Question of Jury Rape Myth Acceptance’ (2023) 7(1) Journal of Gender-Based Violence 189; Joanne Conaghan and Yvette Russell, ‘Rape Myths, Law, and Feminist Research: Myths About Myths?’ (2014) 22(1) Feminist Legal Studies 25; Fiona Leverick, ‘What Do We Know about Rape Myths and Juror Decision Making?’ (2020) 24(3) International Journal of Evidence and Proof 255; Jennifer Temkin and others, ‘Different Functions of Rape Myth Use in Court: Findings from a Trial Observation Study’ (2018) 13(2) Feminist Criminology 205; Ellen Daly, *Rape, Gender and Class: Intersections in Courtroom Narratives* (Palgrave Macmillan 2022); Hannah Bows, *Sexual Violence Against Older People* (Routledge 2019).

58 Bates (n 1); for a discussion on development of sexual history evidence, see Joanne Conaghan and Yvette Russell, *Sexual History Evidence and the Rape Trial* (Bristol UP 2024).

59 Deborah Gorham, ‘“The ‘Maiden Tribute of Modern Babylon’ Re-examined’ (1995) 2193) Victorian Studies 317; Conley (n 55) 122.

60 Lammasniemi, ‘Precocious Girls’ (n 8) 241.

61 Mari Takayanagi, ‘Sacred Year or Broken Reed? The Sex Disqualification (Removal) Act 1919’ (20200 29(4) Women’s History Review 563.

62 Anne Logan, *Feminism and Criminal Justice* (Springer 2008) 140.

63 AMSH, ‘Minutes of the Police Court Rota Sub-Committee’ (11 July 1918) file 3AMS/A/03/08, WL.

64 Bates (n 1) 14.

65 Defendant’s reasonable belief that the complainant was above the age of 16 was a sufficient defence under s 5 CLAA 1885, but there was no defence under s 4, dealing with children under the age of 13.

66 For further discussion, see Jackson (n 55); Bates (n 1).

67 Children Act 1908, s 30.

68 DPP 4/XXX and Calendar of Prisoners, 1868–1929 (X869).

69 House of Lords Criminal Appeal (X869).

70 Crim 1/XXX (X92); MEPO 3/XXX (X862); MEPO 3/XXX (X861).

71 Series of high-profile domestic claims against the defendant (dates of publications removed to ensure anonymity is not compromised through cross-referencing, as per privileged agreements), *Western Daily Press* (Thursday XX/XX 1925); *Western Daily Press* (XX/XX 1920).

72 MEPO 3/XXX (X861), letter 28 June 1928.

73 Defendants in these cases were usually charged with s 62 of the Offences against the Person Act 1861, encompassing both attempted carnal knowledge and/or indecent assault upon a male.

74 Crim 1/XXX (X649); Crim 1/XXX (X313).

75 Crim 1/XXX (X232).

76 In all of the cases reviewed, there was only one case involving a female defendant charged with indecent assault on a boy under the age of consent; all other cases involved male defendants.

77 Offence under the Buggery Act 1533.

78 Jeffrey Weeks, ‘“Sins and Diseases”: Some Notes on Homosexuality in the Nineteenth Century’ (1979) 1 History Workshop 211.

79 For full discussion, see Jeffrey Weeks, *Sex, Politics and Society: The Regulation of Sexuality Since 1800* (4th edn, Routledge 2017).

80 Pseudonyms used throughout; locations and other identifiable data changed.

81 An offence under s 56 of the Mental Deficiency Act 1913.

82 Crim 1/XXX, (X661).

83 Bourke (n 46).

84
*Fletcher* (1859) (n 47) 134; *Fletcher* (1866) (n 48); *R v Barratt* (1873) 12 Cox Crim Cas 498 (Ct Crim App).

85 For a discussion on the history of these frameworks, see Series (n 6) ch 3.

86 Mumm (n 7) 527.

87 Tahaney Alghrani, *Wayward Girls in Victorian and Edwardian England: Pathways In and Out of Juvenile Institutions, 1854–1920* (Bloomsbury Publishing 2024).

88 Rachel Pimm-Smith, ‘District Schools and the Erosion of Parental Rights under the Poor Law: A Case Study from London (1889–1899)’ (2019) 34 Continuity and Change 401.

89 Mumm (n 7) 527.

90 See in particular Bartley (n 7).

91 Jennifer Wallis, *Investigating the Body in the Victorian Asylum: Doctors, Patients, and Practices* (Palgrave Macmillan 2017).

92 Susanna Menis, ‘More Insights on the English Borstal: “Shaping” or Just “Shaking” the Young-Offender? (2012) 5(3) International Journal of Criminology and Sociological Theory 985.

93 Series (n 6).

94 Report of the Royal Commission on the Care and Control of the Feeble-Minded (HMSO 1908) available at <https://wellcomecollection.org/works/j56q4s5z>.

95 For fuller discussions on eugenics, see eg Thomson, Searle, Carey and Mazumdar (all n 10).

96 For fuller discussions on the history of these frameworks, see Series (n 6); Thomson (n 10).

97 HC Deb 10 June 1912, 64.

98 Royal Commission (n 94).

99 Sandland (n 45).

100 Phil Fennell, *Treatment Without Consent Law, Psychiatry and the Treatment of Mentally Disordered People Since 1845* (Routledge 1996).

101 For contemporary eugenicists discussion, see Havelock Ellis, ‘The Sterilisation of the Unfit’ (1909) 1(3) Eugenics Review 203.

102 Thomson (n 10) ch 5.

103 MDA 1913, s 56.

104 Simmons (n 51).

105 MDA 1913, s 56.

106 Thomson (n 10) ch 1.

107 Pamela Cox, ‘Girls, Deficiency and Delinquency’ in Anne Digby and David Wright (eds), *From Idiocy to Mental Deficiency: Historical Perspectives on People with Learning Disabilities* (Routledge 1997) ch 9.

108 Inspection report by L Collard on Dovecot School, Knotty Ash, 26 January 1918, LCC, GLRO EO/PS/12/SP/124/1.

109 Janet Walmsley, ‘Women and the Mental Deficiency Act of 1913: Citizenship, Sexuality and Regulation’ (2000) 28(2) British Journal of Learning Disabilities 45.

110 Crim 1/XXX (X435).

111 ibid.

112 Anna Harpin and Juliet Foster, *Performance, Madness and Psychiatry: Isolated Acts* (Springer 2014).

113 Walmsley (n 109) 45–87.

114 ibid; Thomson (n 10); for further discussion on institutionalisation, see Bartley (n 7); Regina Kunzel, *Fallen Women, Problem Girls: Unmarried Mothers and the Professionalization of Social Work, 1890–1945* (Yale UP 1993).

115 Crim 1/XXX (X465); Crim 1/XXX (X569).

116 Crim 1/XXX (X661).

117 ibid.

118 I could not find any trace of the child, nor any children of women who were detained in colonies in ‘Ancestry’ and other records, implying that the children were in the care of authorities and that the children never lived with the complainants, defendants or their families.

119 The Sexual Offences Act 1956, s 8 is a restatement of the s 56 offence; MDA 1913 was repealed by the Mental Health Act 1959, which aimed to move away from legalism and institutionalisation.

120
*R v C* [2009] UKHL 42, [27].

121 Law Commission, *Consent in Sex Offences: A Report to the Home Office Sex Offences Review* (HMSO 2000) para 4.59. The Law Commission had already conceded that civil law tests should be transplanted to criminal law in *Consent in the Criminal Law* (Law Com CP No 139, 1995), having first considered it in *Mentally Incapacitated Adults and Decision-Making: An Overview* (Law Com CP No 119, 1991).

122 Home Office, ‘Setting the Boundaries Volume I: Reforming the Law on Sex Offences’ (Home Office 2000).

123 Sexual Offences Act 2003, s 75(2).

124 Sexual Offences Act 2003, ss 38–41 on care workers for persons with a mental disorder; ss 25 and 26 on familial child sex offences; ss 16–19 on abuse of position of trust.

125 The Sexual Offences Act 2003, s. 30(2) describes it as follows: ‘he lacks the capacity to choose whether to agree to the touching (whether because he lacks sufficient understanding of the nature or reasonably foreseeable consequences of what is being done, or for any other reason)’. Subsections s.31(2); s.32(2) and s.33(2) use the similar wording to cover instances of engaging in sexual activity, being present during a sexual activity and watching a sexual act, respectively..

126 For legal rules on intoxication, see *Bree* (n 30); for a discussion, see Cowan (n 12) 899.

